# Influence of Material Degradation on Deformation of Paraglider during Flight

**DOI:** 10.3390/ma16155396

**Published:** 2023-08-01

**Authors:** Paulina Maślanka, Halina Szafrańska, Andrii Aleksieiev, Ryszard Korycki, Patrycja Kaziur, Anna Dąbrowska

**Affiliations:** 1 Interdisciplinary Doctoral School, Lodz University of Technology, 90-924 Lodz, Poland; andrii.aleksieiev@dokt.p.lodz.pl (A.A.); patrycja.kaziur@dokt.p.lodz.pl (P.K.); 2 Department of Physicochemistry and Materials Technology, Faculty of Chemical Engineering and Commodity Science, Kazimierz Pulaski University of Technology and Humanities in Radom, 26-600 Radom, Poland; h.szafranska@uthrad.pl; 3 Department of Mechanical Engineering, Informatics and Chemistry of Polymer Materials, Lodz University of Technology, 90-924 Lodz, Poland; ryszard.korycki@p.lodz.pl; 4 Department of Personal Protective Equipment, Central Institute for Labour Protection—National Research Institute (CIOP-PIB), 90-133 Lodz, Poland; andab@ciop.lodz.pl

**Keywords:** material aging, aerodynamic characteristics, strength characteristics, air permeability, numerical modeling

## Abstract

The aim of this article is to determine experimentally and numerically the influence of material degradation on the deformation of a paraglider during flight. The presented method regards numerical modeling of pressure distribution over the wing and its effect on paraglider behavior; the considerations are preceded by experiments on three types of Polyamide 6.6 paraglider fabrics, subjected and not subjected to thermal, UV and flexing degradation. Scanning electron microscope (SEM) records allowed to determine the structural characteristics of the analyzed samples. Air permeability and mechanical tests are the input data for the computational simulations. When a pressure drop of 200 Pa is applied, all the analyzed samples are impermeable, except for those damaged by flexing. Thus, flexing damage has the greatest influence on the air permeability change among all considered aging factors. Aging caused by UV radiation has the greatest influence on mechanical properties. No major influence of thermal ageing on the mechanical properties of the considered samples is observed. Safety factors of the considered materials not subjected to degradation range between 3.94 and 6.00. Safety factor of fabric no. 1 subjected to the UV degradation is equal to 1.33; this result does not secure a safe usage of the considered material. The methodology described in this research can help to predict paraglider covering materials’ behavior in flight; it assumes many cases, i.e., applying a new material or the material at any point of its life cycle. Thus, the practical implications of this model supported by numerical methods may result in saving time and cost in producing prototypes, as well as potentially assessing the safety of used wings. Future research activity can introduce the application of different elastic–plastic damage models to determine the paraglider behavior during collapse.

## 1. Introduction

Paragliders are devices intended for gliding; their wings, of an elliptical outline, are manufactured from the following: upper and lower panels, and ribs. All of the segments are woven fabrics connected by seams [1,2]. Therefore, the wings do not have any rigid elements applied. The other basic elements of a paraglider are lines, risers, and the harness [1]. 

Deformation of the materials caused by the overpressure of air acting from the inside of the wing can influence the decrease in the aerodynamic characteristics. Therefore, in addition to the basic requirements of the paraglider/parachute fabrics (i.e., good relation of mass to strength, low thickness, air permeability parameter tending towards zero), the materials should also be possibly stiff [1,3]. 

In order to uncover important information about the structure and filaments/yarns forming the analyzed woven samples, scanning electron microscopy (SEM) is used [4,5,6,7].

The influence of the deformation of the wing on the aerodynamic characteristics of a paraglider has been studied by some researchers. 

For example, in [8], tests were performed of single paraglider cells in a wind tunnel in configurations, namely, a deformable paraglider cell and a rigid cell mock-up. 

A simplified parachute cell mock-up was studied in [9] in order to evaluate the deformation and internal pressure. Rigid and deformable cells were compared in [10] in order to assess different configurations of the applied geometry and arrangements of inlets.

Let us mention that paraglider fabrics are also highly exposed to external factors, such us UV radiation, gusts or moisture [1,11,12]. Multiple unpacking and packing of the paraglider wing can cause mechanical degradation, whereas improper storage in temperatures that are too low or too high can also have unfavorable influence on the material properties of the wing. Therefore, paraglider fabrics are required to be resistant to degradation.

There are many kinds of flexing fatigue resistance test methods, such as the De Mattia method, the Schildknecht method, the crumple/flex method, etc. [13,14,15,16,17]. Since the methods differ from each other in principle, the results obtained from them cannot be compared.

The UV, thermal and mechanical degradation can possibly cause an increase in the air permeability values and decrease in the mechanical properties, which is studied further in this work. 

The values obtained in the experimental analysis are the input data for the numerical modeling section. 

This work is based on two types of numerical calculations: the computational fluid dynamics (CFD) based on the finite volume method and the structural calculations based on the finite elements method (FEM). 

The advantages of the numerical methods include [18,19,20]: (1) visualization of the obtained results and clear information about variables related to the behavior of the object; (2) ability to easily make geometrical changes on the virtual model and analyze the impact of these changes on the obtained results/operation of the object (paraglider sensitivity to these changes); (3) omission of problems related to creating real conditions (i.e., very high velocities, high/low temperatures, large loads acting on materials) in research centers/laboratories.

Numerical calculations of the flow over the paraglider wing were previously performed in the sense of geometry influence [21,22] or the position of the inlet to the airstreams [23] on the aerodynamic characteristics, whereas the previous research of the authors of this publication studied the sensitivity of paraglider performance to air permeability material change [24].

The numerical calculations regarding the structural behavior of the materials were not found in the literature. Most of the research focused on the wing deformation were based on the wind tunnel trials or observations of the real flight conditions [8,9,10,25].

The first step of modeling the deformation is obtaining the pressure distributions over the wing covered with materials of different air permeability values in the computational flight simulation. 

The pressure difference between two sides of the paraglider fabric is, in fact, pressure acting on the material. Thus, the pressure distribution, together with the experimentally obtained mechanical properties of fabrics under consideration, are the input data for the structural analysis. 

Based on the above, the following studies were performed: (1) basic material properties determination; (2) woven structure analysis based on the scanning electron microscope (SEM) records; (3) influence of the UV, thermal and flexing damage on the air permeability and mechanical properties of the paraglider fabrics; (4) CFD numerical determination of the pressure distribution over the paraglider wing geometry; (5) FEM structural analysis of the considered materials subjected and not subjected to the degradation.

The topic regarding development of the paragliders connects many fields, such as materials engineering, aerodynamics and flight mechanics. Thus, the novelty aspect of the work is the original multidisciplinary application and synergy of these elements in paraglider design.

The article is focused on an experimental and numerical study of the influence of material degradation on the deformation of a paraglider during flight. It is a continuation of the previous study regarding the experimental and numerical determination of strength characteristics related to a paraglider wing with Fourier transform infrared spectroscopy of applied materials [26]. The previous studies did not consider predicting the paraglider covering degraded materials’ behavior in flight, which can be helpful in the initial assessment of safety of a used paraglider or speculating its life cycle length. The proposed model can also be a base for studying other objects, such as parachute drops carrying high loads. The obtained results can be part of the optimization procedure for the paraglider flight conditions at the lower material volume/weight limit. However, this concept requires further development of the optimization functional [27,28] and structural analysis in the micro scale [29,30]. 

## 2. Materials

The analyzed materials are three PA 6.6 coated with polyurethane resins paraglider fabrics characterized by the standard ripstop weave. The samples were received thanks to a manufacturer from Poland. The authors were obliged not to mention the trade names of the samples nor the manufacturer. 

Obtained surface masses of the fabrics are in the range of 26–42 g/m^2^ and their thicknesses are in the range of 0.05 mm–0.09 mm (Table 1). Depending on the composition of the impregnation, it may influence its final density. Therefore, the higher mass of the final product does not always indicate greater thickness of it (see samples 2 and 6); the exact chemical groups were studied in previous research [26].

All the samples are of air permeability tending towards zero, when not subjected to degradation, which is further studied in the subsequent sections.

## 3. Methods

### 3.1. Experiments

All the laboratory tests were performed in the normal climate conditions, i.e., *T* = 20 °C; *p* = 1013.25 hPa; *RH* = 65%, unless otherwise indicated.

#### 3.1.1. Scanning Electron Microscope (SEM)

A Prisma^TM^ E Scanning Electron Microscope (Thermo Fisher Scientific, Waltham, MA, USA) was used in order to record pictures of magnitudes equal to 120×, 200× and 500×. In order to analyze woven structure of the considered fabrics, a magnitude of 40× was also applied. The pictures of the lower magnitude were not placed in this publication; however, some observations based on them were described below in Section 4.1. 

#### 3.1.2. UV Degradation

A paraglider wing is highly exposed to UV radiation. Therefore, the samples were artificially aged in the QUV Accelerated Weathering Tester aging chamber AATCC TM 186 (Q-Lab Corporation, Cleveland, OH, USA). The samples were irradiated in conditions similar to natural ones for a temperate climate according to the PN EN ISO4892-3 [31] standard, based on the Technical Report TR 010 ed. May 2004 “Exposure procedure for artificial weathering”. The sources of UV radiation were UVA-340 fluorescent lamps equivalent to midday sunlight (months: June, July). The following conditions were applied: intensity of 0.76 W/m^2^ (measured with lambda = 340 nm); temperature of 60 °C; time of performing the test, 72 h; relative humidity of 65%.

#### 3.1.3. Heating

High temperatures of 50 °C, 60 °C and 70 °C were applied in the time cycles of 24 h, 48 h and 72 h. The test was performed using the SBS-ADO-2000 2170 W 136 l (Steinberg Systems, Berlin, Germany) heating device. The method of degradation was chosen to simulate the conditions of improper storage of paragliding equipment, e.g., storing the equipment in a car trunk during a hot and sunny summer. 

#### 3.1.4. Freezing

A Stirling SU780XLE (Biolife Solutions Inc., Bothel, WA, USA) device was used in order to subject the considered samples to a low temperature equal to −30 °C in a time of 24 h. Paraglider/parachute materials can be subjected to low temperatures, for example, when improperly stored or during high-altitude parachute opening.

#### 3.1.5. Flexing Damage

Damage by flexing was applied according to EN ISO 7854 [14] Standard, Method C (i.e., crumple/flex method). In this method, rectangular samples (of dimensions of 220 mm × 190 mm) were sewn into a cylindrical shape, where the diameter of each shape was equal to 64 mm and its height to 190 mm. The cylindrical coated sample was placed between 2 moving discs. One of the discs caused twisting of the fabric sample (by rotating on its axis; 200 twists/min), whereas the other disc compressed the fabric by a pushing motion (152 strokes/min). These simultaneous actions were repeated 9000 times. The test was performed using Crumple Flex Tester TF117C (Testex, Dongguan, China).

#### 3.1.6. Color Stability

A Conica Minolta CM-3600d spectrophotometer (Sony, Tokyo, Japan), with a spectral measuring range of 360 nm–740 nm, was used to analyze the effect of degradation factors on the color stability of aged and unaged samples. In accordance with the PN-EN ISO 105-J01 [32] standard, the change in color and brightness was determined in accordance with the CIE-Lab color space of Equation [33]:

(1) $$dE_{\mathrm{ab}}^{*}=\sqrt{{\Delta a}^{2}+{\Delta b}^{2}+{\Delta B}^{2}},$$

where

$dE_{\mathrm{ab}}^{*}$—difference between two colors in L*a*b* color space [-]

Δ*a*—deviation from the color of the reference sample in the axis of red-green [-]

Δ*b*—deviation from the color of the reference sample in the axis of yellow-blue [-]

Δ*B*—deviation in the brightness parameter from the color of the reference sample [-]

#### 3.1.7. Air Permeability

The air permeability parameter of the considered samples was tested using an FX 3300 (TEXTEST AG, Schwerzenbach, Switzerland) digital device. The determination of it was based on the EN ISO 9237:1995 standard [34], where the pressure equal to 100 Pa is usually applied. However, paraglider fabrics’ manufacturers describe the air permeability with a pressure drop of 2000 Pa. Moreover, after applying initial flow over a paraglider wing geometry, it was found that the actual pressure drop acting on the paraglider material in normal flight conditions is around 200 Pa.

In order to obtain precise results of the air permeability parameter, in the below analysis, the following pressure drops were applied: 200 Pa, 1500 Pa, 2000 Pa and 2500 Pa. A total of 10 measurements were taken per each sample/each condition. 

#### 3.1.8. Tensile Properties

The tensile properties of fabrics not subjected and subjected to aging were determined using an Instron device and according to the EN-ISO 13934 [35] standard. A total of 20 measurements were taken for each sample, 10 for each direction. The measurement error for the selected samples was less than 3%. The width of the samples was equal to 50 mm, the distance between clamps was 200 mm and the speed of tensile was 100 mm/min.

### 3.2. Simulations

The computational fluid dynamics (CFD) based on the finite volume method ANSYS Fluent 2022 R2 solver was used to determine the pressure distribution over the paraglider wing; stress, strain and deformation distributions over 3D model of a paraglider were determined using the ANSYS Structural program and the finite elements method (FEM).

The finite volume method allows the use of non-orthogonal, non-uniform computational meshes. It assumes that the fulfillment of mass, momentum and energy balances for each elementary cell is tantamount to the fulfillment of these balances in the entire considered space. Thus, non-linear differential equations that describe the conservation of mass, momentum and energy are numerically solved [19]:

(2) $$\begin{array}{cc} & {∭}_{V}^{\;}\frac{\partial_{\rho }}{\partial_{t}}dV+\iint_{A}^{\;}\rho v_{n}dA=0\\ & \frac{d}{dt}{∭}_{V}^{\;}\rho \vec{v}dV=\iint_{A}^{\;}{\vec{p}}_{n}dA+{∭}_{V}^{\;}{\vec{F}}_{m}\rho dV\\ & \frac{d}{dt}{∭}_{V}^{\;}\rho (c_{w}T+\frac{v^{2}}{2})d=\iint_{A}^{\;}{\vec{p}}_{n}\cdot \vec{v}dA+{∭}_{V}^{\;}\rho {\vec{F}}_{m}\cdot \vec{v}dV+\iint_{A}^{\;}{\dot{q}}_{n}dA+{∭}_{V}^{\;}{\dot{q}}_{m}\rho dV \end{array}$$

where

V—volume of an area bounded by a closed surface A [m^3^]

ρ—Fluid density [kg/m^3^]

$\vec{v}$—Velocity vector [m/s]

$\vec{p}$—Surface stress vector [Pa]

${\vec{F}}_{m}$—Vector of body forces [N/m^2^]

$c_{v}$—Specific heat [$\frac{J}{kg\cdot K}]$

T—Temperature [K]

${\dot{q}}_{n}dA$—Heat flux density related to specified surface [$\frac{J}{m^{2}\cdot s}]$

${\dot{q}}_{m}\rho dV$—Heat flux density related to specified volume [$\frac{J}{m^{3}\cdot s}]$

The Finite Element Method (FEM) uses both linear and non-linear partial differential equations to be solved in the following iterations. The principle of this method is to solve governing equations concerning the following: (1) assuming the local equilibriums; (2) strain–displacement relations; (3) predicting the material’s response to external factors acting on it, i.e., constitutive equations. All the equations are solved with the assumption of previously defined boundary conditions based on the real physical conditions [18,19].

The solution presented below is based on the Newton–Raphson method. This method is implemented when the force–displacement curve is non-linear. First, the system is stationary. Next, consecutive iterations of displacements and forces are implemented until the convergence is achieved. 

The Newton–Raphson procedure can be described by the following formulas [20], which regard displacement increment and residual force:

(3) $$\begin{array}{cc} & ∆u^{n}=(F_{ext}-F_{int}^{n})/K^{n}\\ & F_{res}^{n}=F_{ext}-F_{int}^{n} \end{array}$$

where

*n*—Iteration (subsequent) [-]

*F_ext_*—Applied force [N]

*F_int_*—Computed internal force [N]

*F_res_*—Residual force [N]

Δ*u*—Displacement increment [m]

*K*—Stiffness matrix [Pa]

The convergence is achieved when the residual force in the current step of calculations is not greater than the assumed difference between the applied and calculated force. Thus, the residual value satisfies the convergence criteria.

## 4. Experimental Results

### 4.1. Scanning Electron Microscope (SEM)

**Figure 1:**
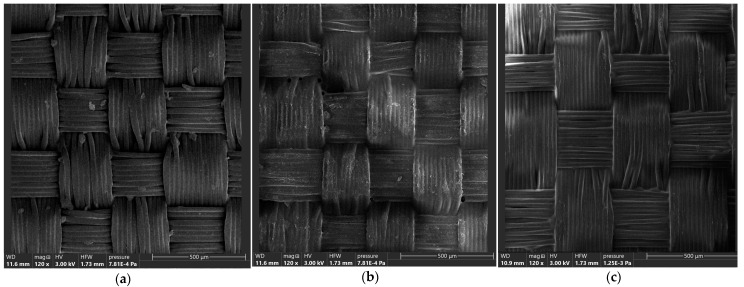
Scanning electron microscope records of the considered samples. (**a**) sample no. 1, (**b**) sample no. 2, (**c**) sample no. 6.

According to the SEM records presented in Figure 1, all the considered woven fabrics were manufactured using multifilament yarns. No twist of the yarns was observed. Depending on the type, surface mass and weave of fabric, the diameter of the filaments and yarns differ, which will be discussed further in this section. 

There is a high correlation between the structural characteristics of yarns and woven fabrics, which has been studied widely by many researchers. The most important studies in this area were performed by Peirce, Dastoor, Kemp or Olofsson, etc.; they created models containing formulas, which correlated both structural and mechanical properties of yarns and fabrics [36,37]. 

All the filaments that can be seen in Figure 1 were significantly flattered. This is a result of high values of weave factor and yarn extension, low crimp factor, as well as no/insignificant twist of the yarns. The fabrics had probably been also calendared during the manufacturing process. Both tight weave and calendaring result in achieving the required final parameters of the fabric, which is mainly air permeability tending towards zero. A high density of threads per width results in the increased mechanical properties, whereas calendaring flatters the final product, which enables better packing properties (when a paraglider is considered, it would be decreased volume of a wing). 

Fabrics weaves were studied with the magnitude of 40×; however, the records compiled in Figure 1 also present some characteristic elements of it. Every sample is characterized by thicker and thinner yarns. The thicker yarns represent the previously mentioned reinforcement characteristic for the ripstop weave. Based on the obtained pictures and organoleptic analyses, it was noticed that all the considered fabrics presented the traditional ripstop of squares pattern. In each considered case, reinforcement contained two thicker threads/1 edge. The exact number of thinner threads/1 edge was compiled in Table 2.

**Figure 2:**
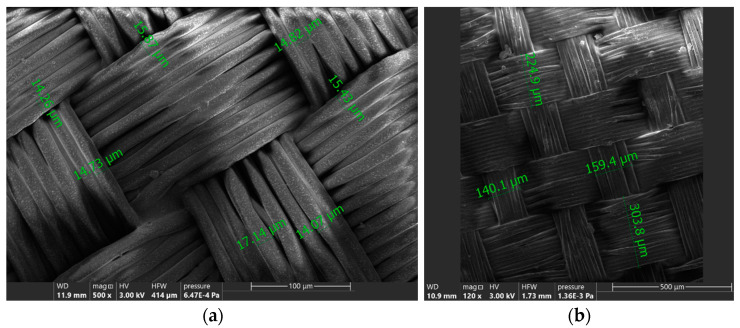
Example of determination of diameters of (**a**) filaments (sample 1) and (**b**) yarns (sample 6).

Based on the pictures achieved by the SEM device, diameters of the single filaments, as well as threads, were determined, as shown in Figure 2. An amount of 20 measurements per each parameter of each sample were taken. The results were compiled in Table 2.

Based on Table 1 (Section 2) and Table 2, it can be concluded that obtaining thinner fabric is highly correlated with adjusting thinner filaments. However, it does not have correlation with the diameter of yarn, as the yarns in this type of fabrics are significantly flattered in the final product. 

Even though the warp and weft diameter of thin and thick yarn is higher for sample 6, its filament diameter is less than that of sample 2. It may be a result of more compressible positioning of the filaments in the yarn in sample no. 2.

**Figure 3:**
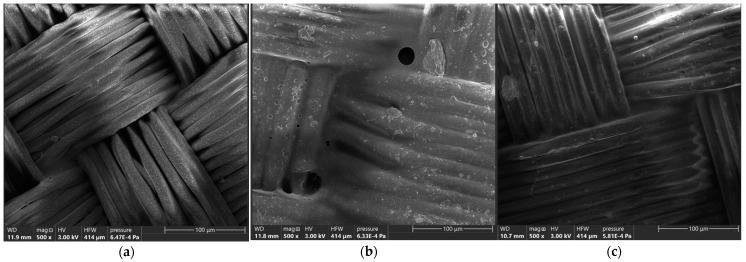
Impregnation on the analyzed samples. (**a**) sample no. 1, (**b**) sample no. 2, (**c**) sample no. 6.

When an increased magnitude of 500× was used, the impregnation was clearly visible, as seen in Figure 3. The polyurethane resins not only glued the spaces between interlacements, but also those between filaments. Therefore, the coverage of the fabrics causes the achievement of air impermeability when testing even under relatively high pressure drops (2000 Pa). Depending on the model and type of the fabric, the amount of impregnation and its general picture differed from each other.

### 4.2. Influence of Aging on the Material Properties

#### 4.2.1. Color Stability

**Figure 4:**
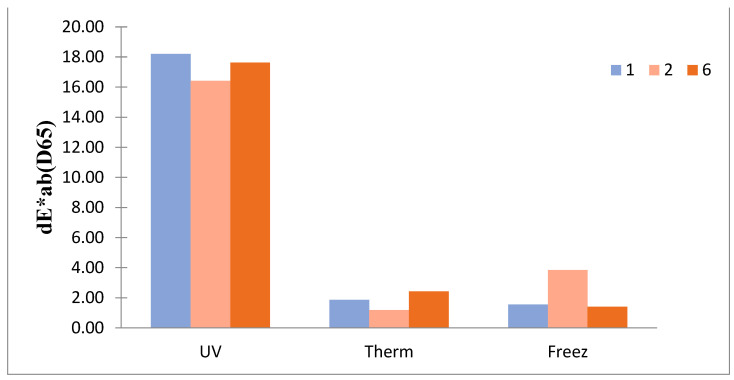
Color change of the analyzed samples to the UV, thermal (heating) and freezing aging.

The visible, and sometimes invisible, alteration of the outward appearance can be determined directly by measuring the color change ($dE_{\mathrm{ab}}^{*}$) of the surface. This is one of the most important measurements as the aging begins at the surface of materials. The results obtained during the color change test are presented in Figure 4.

Ultraviolet aging caused the greatest color change for all the considered samples, reaching the value of 16.42–18.21.

It was observed that both the high and low temperatures did not have a noticeable influence on the color of the tested samples when organoleptic evaluation was performed. The results obtained by spectrophotometer and compiled above confirm this observation. The obtained values of color change ranged between 1.19 (sample no. 2, heating) and 3.85 (sample no. 2, freezing). According to the literature, the tolerance of the $dE_{\mathrm{ab}}^{*}$ is usually 5 [38].

The reason for the obtained results is that Polyamide 6.6 is susceptible to UV degradation, including decomposition of the functional groups [26], whereas it is not as susceptible to degradation caused by high and low temperatures at values considered in this study.

#### 4.2.2. Air Permeability

The obtained results of air permeability parameter were compiled in Table 3 below.

**Figure 5:**
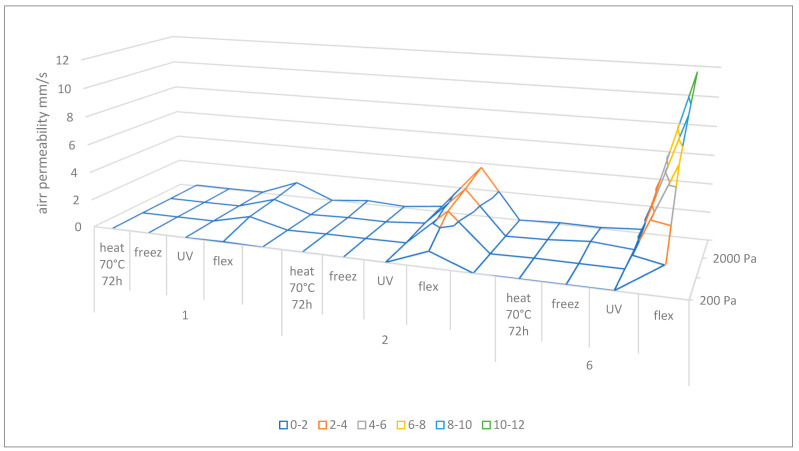
Air permeability parameter of samples 1, 2, 6 subjected to heating (70 °C, 72 h), freezing, UV aging, flexing damage.

According to Table 3 and Figure 5, when a pressure drop of 200 Pa was applied, only samples damaged by flexing were permeable. Thus, flexing damage has the greatest influence on the air permeability change among all considered aging factors. 

For fabrics no. 1 and 2, UV degradation had a higher influence on the air permeability change compared to temperature factors. However, when sample no. 6 was considered, freezing and UV degradation had comparable effect on this parameter. 

When increased pressure drops were applied (1500 Pa–2500 Pa), the air permeability values of samples subjected to all types of degradation were greater than zero. However, it did not apply to all temperatures and time cycles of heating. 

**Figure 6:**
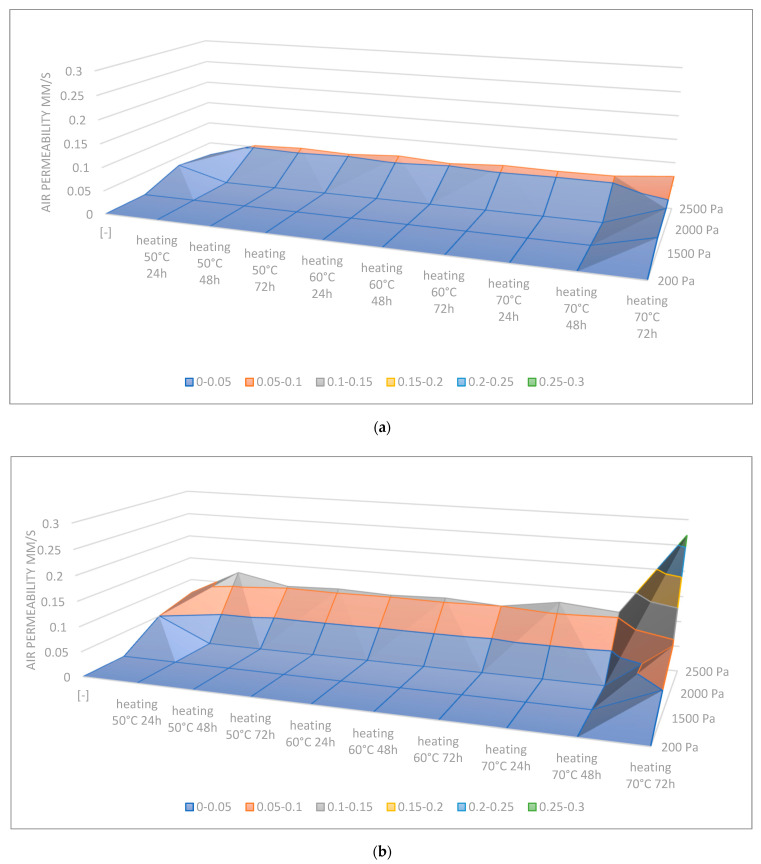

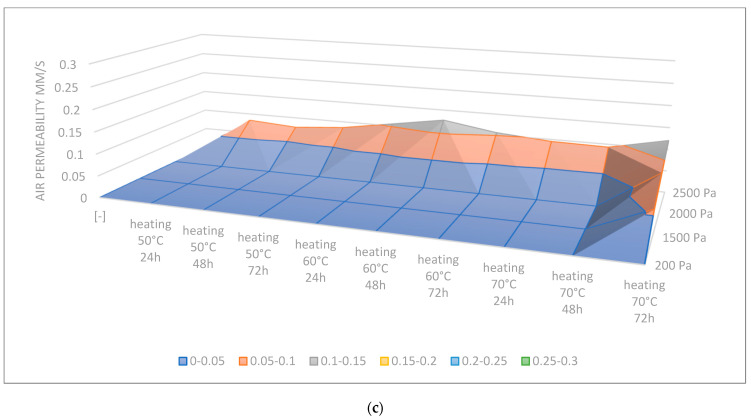
Surface charts of the air permeability change in the function of time + temperature and pressure drop acting on material: (**a**) sample no. 1; (**b**) sample no. 2; (**c**) sample no. 6.

Surface charts presenting the dependences of air permeability values from the applied times and temperatures of heating, as well as the pressure drops, were compiled in Figure 6 below. 

Based on the charts, it can be concluded that the most susceptible to this type of aging was material no. 2, whereas the least visible changes in the air permeability value were observed in the case of sample no. 1. Increasing the time and temperature of heating, as well as the pressure drop, has a significant impact on the obtained permeability values. 

Differences in permeability result from the composition and modification of the considered samples. All the samples indicated greater values of air permeability under the influence of the flexing method. It is caused by mechanical destruction of the structure of materials in the considered method. On the other hand, classic aging methods, i.e., UV degradation, heating and freezing, have a slightly different effect on the material itself; for example, the UV influence is focused on the surface of the material, and the other two directly on the material itself as a whole. The aging methods focus on the destruction of functional groups. That is the reason why there are such differences in the aging influence on the air permeability values. Of course, the changes are also dependent on the quantitative components (phr) of the matrix and the reinforcement.

#### 4.2.3. Tensile Properties

**Figure 7:**
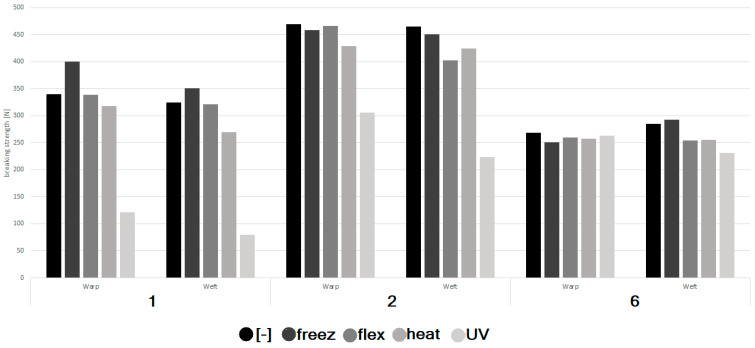
Graph describing the maximal forces during elongation of samples no. 1, 2, 6 not subjected ([-]) and subjected to aging: freezing (freez), flexing (flex), heating (heat) and UV radiation.

The tensile test results were compiled in Table 4 and in Figure 7 below. 

According to the results compiled in Table 4 and Table 5 and Figure 7, aging caused by the UV radiation had the greatest influence on mechanical properties. It was observed especially when samples no. 1 and 2 were considered (decrease in warp, respectively, 64% and 35%; in weft, respectively, 75% and 52%). In the case of sample no. 6, a decrease in the tensile properties was observed; however, it was insignificant, i.e., 2% (warp) and 19% (weft). 

No significant influence of freezing on the mechanical properties of the considered samples was observed. Moreover, in the case of sample no. 1 subjected to freezing, an increase in the obtained values was noticed. The coefficient of variation of tests regarding sample no. 1 not subjected to aging and subjected to freezing was around 3 N, when tested into the warp direction, and around 20 N, when tested into the weft direction. Thus, it can be concluded that the results reflected the actual state. 

Flexing damage was expected to have a great influence on the decrease in mechanical characteristics of the considered samples, as it causes significant mechanical damage of threads and impregnation (the air permeability results confirm the above). However, the tensile results did not show this dependence. This is probably caused by the dimensions of the tensed sample, which significantly exceeded the area damaged by flexing. In order to find the actual dependence between flexing damage and breaking force, a single yarn should be subjected to the test. However, limited access to the fabric samples prevented verification of this thesis.

Heating had a noticeable, but not significant, influence on the mechanical characteristics of the tested samples. The decrease in maximal force during elongation was between 6 and 10% compared to the samples not subjected to aging. 

The graphs recorded during the tensile tests allowed to determine the Young’s modulus values of the samples subjected and not subjected to aging. According to [26], which can be considered as an introduction to this publication, only the linear part of the curve of load to extension was used to the calculation of the Young’s modulus value. 

The Young’s modulus values that were applied to the analyzed fabrics were determined according to the above and the scheme described in [26]. They are listed in Table 5 below.

Due to the anisotropic character of woven fabrics, the values of linear elasticity differed when both directions of one type of a fabric were considered.

## 5. Simulations

### 5.1. Modeling of Paraglider Wing

As this publication is a continuation of previous research of the authors, CFD and FEM models were designed according to the exact steps from [24,26]. 

**Figure 8:**
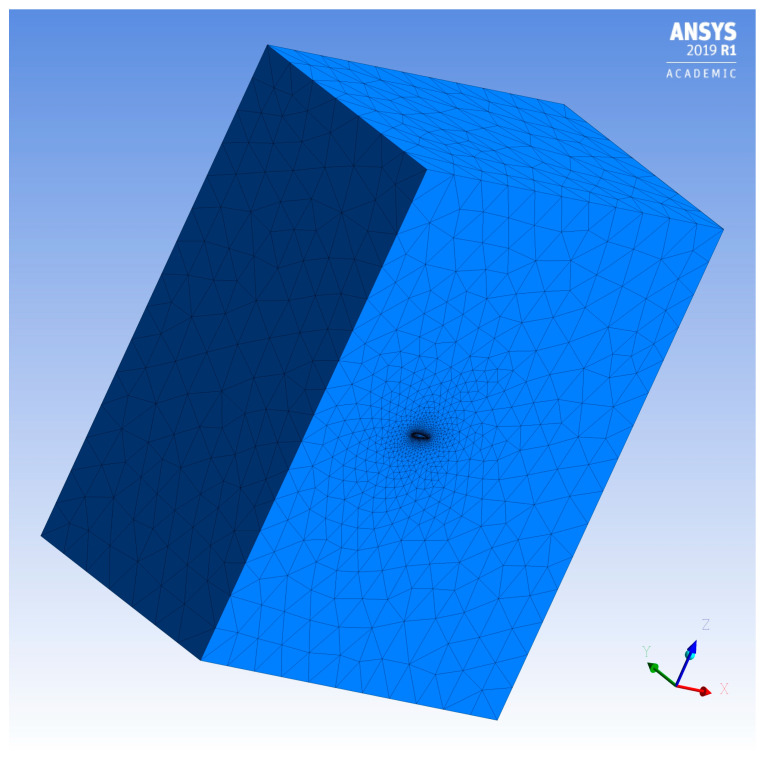
The finite volume mesh intended for the CFD calculations.

The two methods require different types of meshes and the application of boundary conditions. Thus, the paraglider wing geometry dedicated for the finite volume method was surrounded by the calculation area. The outer surfaces of the calculation area represented the pressure-far-field condition (undisturbed flow). In order to control the change of the flow over the whole area, it was fully filled with the finite volume mesh elements, as seen in Figure 8.

**Figure 9:**
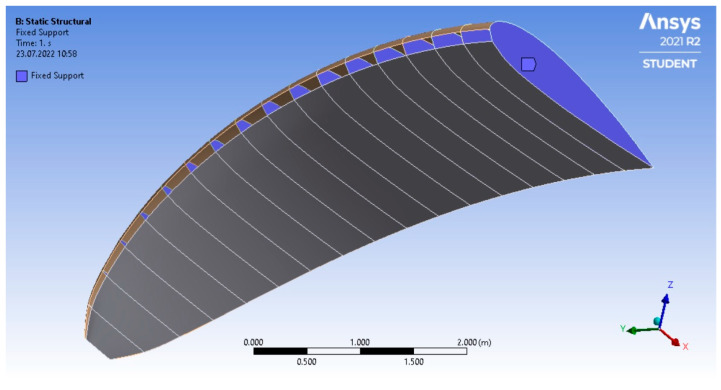
The paraglider model intended for the FEM structural calculations.

The FEM model required only the shell type geometry of the wing including the ribs, which corresponded to the fixed support boundary condition, as seen in Figure 9. 

### 5.2. Numerical Simulations

Based on the previous research of the authors, in the normal and undisturbed flight conditions, a maximal pressure acting on the material (which indicates the pressure difference between the two sides of the considered material) is around 200 Pa. 

Therefore, when studying the influence of the material air permeability parameter on aerodynamic characteristics of the paraglider wing, all the samples that obtained the result of 0 mm/s in the air permeability test when a pressure drop of 200 Pa was applied can be considered as impermeable. 

According to the results compiled in the above Section 4.2.2, all the studied samples not subjected to aging and those subjected to UV and thermal degradation were considered to be impermeable to normal flight conditions, whereas those subjected to the flexing damage were assumed as permeable. 

However, as the flying object can also be subjected to increased overloads, the structural calculations are performed for the more extreme conditions. The overloads can be caused by maneuvers of a pilot, gusts, etc. All the aforementioned can affect the pressure distribution and thus, the tensions inside the considered materials. 

When the most extreme cases are analyzed, the pressure acting on a material during the normal flight conditions is multiplied by a maximal possible overload, which is assumed as 5.6 [26]. Thus, the maximal pressure acting on a material is now assumed to be over 1000 Pa. For the further structural calculations, the reference air permeability values were chosen to be these tested with the pressure drop of 1500 Pa. 

**Figure 10:**
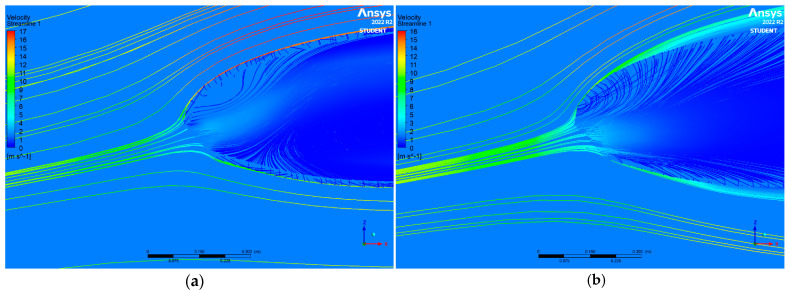
Streamlines in proximity of paraglider inlet: (**a**) fabric no. 1 subjected to flexing damage; (**b**) fabric no. 6 subjected to flexing damage.

As it can be seen in Figure 10, the higher the air permeability value parameter of the considered material, the more significant the air particles permeating through the paraglider covering material.

Based on the picture, it can be observed that the porosity of the material caused the particles to permeate through the paraglider coverage. However, in the first case, it was insignificant and almost did not influence the streamlines’ track around the paraglider on the lower and the upper surface. Streams of air of an increased velocity were observed on the upper surface boundary layer (in the immediate vicinity of the contact boundary between the material covering and the surrounding air, as seen in Figure 10a), whereas the increased permeability of fabric no. 6 subjected to flexing damage disturbed the track of streamlines around the wing. Increased velocity streamlines appeared at a greater distance from the upper surface; moreover, the velocity was decreased compared to the previous case, as shown in Figure 10b. Thus, it had an effect of decreasing the lift force and increasing the drag force values. 

**Figure 11:**
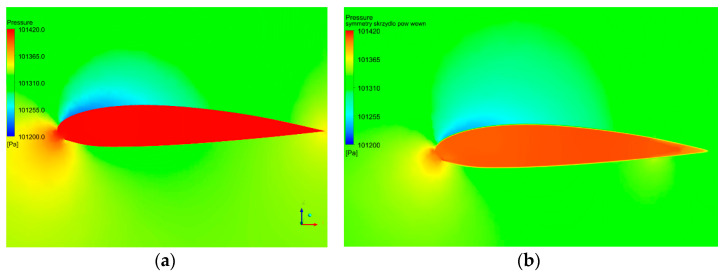
Pressure distribution over paraglider covered with (**a**) air impermeable material and (**b**) material no. 6 subjected to flexing damage.

The more intense air particles permeating also caused the decrease in the pressure difference between two sides of the covering material, as seen in Figure 11 and Table 6. 

The pressure distributions compiled in Table 6 refer to the symmetry plane. Therefore, it should be considered that a paraglider wing in a top view is of an ellipse shape; thus, chord size decreases when approaching the side edge of the wing. Therefore, the pressure values and the distances corresponding to them were recalculated for each segment forming the paraglider.

The material properties described in Section 2 and Section 4.2.3 were the input data for the covering materials in the model under study in the ANSYS Static Structural program, as in [26].

## 6. Simulation Results

**Figure 12:**
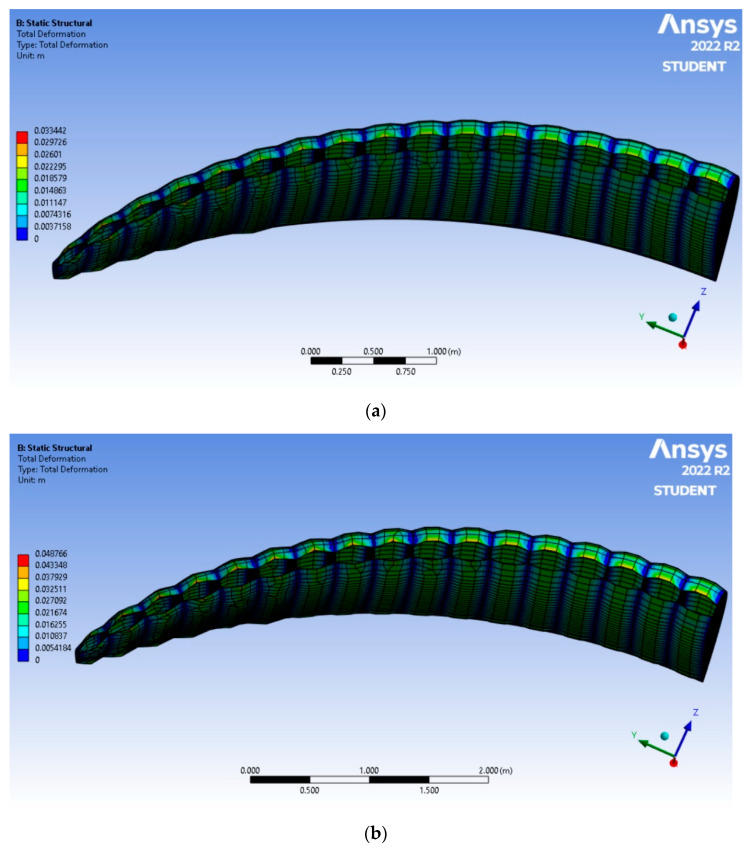
Deformation comparison of materials covering the considered paraglider: (**a**) sample no. 2 not subjected to aging; (**b**) sample no. 1 subjected to the UV aging.

The course of numerical calculation allowed to obtain numerical (Table 7) and visual (Figure 12) results regarding deformation, stress and strain of the considered woven fabrics.

According to Table 7, deformation and strain decreased with the increasing of tensile strength of a material and/or decreasing of pressure acting on a material. The lowest deformation and strain values were achieved for material number 2 not subjected to the aging, and materials no. 2 and 6 subjected to the flexing damage.

According to the results described in Section 4.2.3, the tensile properties of sample no. 2 were the greatest among all the considered fabrics. The deformation and strain values representant of this sample were achieved with the greatest pressure acting on the material, as seen in Table 6.

Maximal pressure values acting on materials no. 2 and 6 subjected to the flexing damage were significantly decreased due to their increased air permeability parameters. Thus, it was possible to obtain insignificant values of stress and deformation.

Sample no. 1, which was characterized by the lowest thickness, obtained the highest stress values among all the materials.

When the tensile properties of this material were decreased (UV degradation), the maximal and average values of stress also decreased.

Based on the above obtained maximal stress results and material characteristics, safety factors for all the considered cases were calculated, according to the formula below; the results were compiled in Table 8 below.

(4) $$f_{s}=\frac{\sigma_{Bmax}}{\sigma_{Cmax}}$$

where:

*f_s_*—safety factor [-]

*σ_Bmax_*—maximal stress at break [Pa]

*σ_Cmax_*—maximal stress obtained in the course of numerical calculations [Pa]

**Table 8:** Safety factors of degraded and non-degraded paraglider materials.

Sample	Safety Factor
1	4.48
2	6.00
6	3.94
1 (heating)	3.75
2 (heating)	5.57
6 (heating)	3.65
1 (UV degradation)	1.33
2 (UV degradation)	2.91
6 (UV degradation)	3.19
1 (freezing)	4.83
2 (freezing)	5.86
6 (freezing)	4.06
1 (flexing)	4.43
2 (flexing)	5.47
6 (flexing)	4.70

Based on the safety factor numerical values listed above, which were obtained by the stress parameter, it was initially concluded that all the considered materials, including these subjected to aging, allowed a relatively safe use of a paraglider.

However, the safety factor equal to 1.33 (sample no. 1 subjected to the UV degradation) is an insignificant number. Thus, the other obtained numbers were studied. The obtained maximal strain of the considered material was equal to 9.1%, which is a greater value than the elongation at break of this material (8.5%). Although the implemented overload was very high (5.6) and the maximal obtained values of strain rarely occurred when its distributions were considered, a paraglider covered with an UV degraded material no. 1 should not be allowed to be used.

The obtained results differed mainly by the values; the behaviors of the stress, strain and deformation distributions were alike in the previous research [26], i.e., the stress distribution differed compared to the deformation and strain distributions. The greatest stress was concentrated in a material in the immediate vicinity of the ribs (rigid support). When deformation and strain were considered, their biggest values were noticed on the leading edge and in the regions between supporting ribs.

In Figure 12 below, the deformation distributions of two materials covering the considered paraglider were compiled: a, sample no. 2 not subjected to degradation (the lowest deformation among all the considered cases); b, sample no. 1 subjected to the UV degradation (the greatest deformation among all the considered cases). The difference is clear and visible organoleptically.

## 7. Conclusions

The aim of this paper was to determine experimentally and numerically the influence of material degradation on the deformation of a paraglider during flight.

Based on the SEM records, the paraglider fabrics are manufactured using multifilament yarns. A significant flatter of the yarns is a result of calendaring of the analyzed fabrics, as well as tight weave. This results in achieving the required final parameters of the fabric, which is mainly air permeability tending towards zero.

Based on the records of the magnitude of 500×, the polyurethane resins not only glue the spaces between interlacements, but also those between filaments. Depending on the model and type of the fabric, the amount of impregnation and its general picture differs from each other.

When a pressure drop of 200 Pa is applied, all the analyzed samples are impermeable, except for those damaged by flexing. Thus, flexing damage has the greatest influence on the air permeability change among all considered aging factors.

Aging caused by UV radiation has the greatest influence on mechanical properties. The greatest decrease in the breaking strength is 75%, when sample no. 1 is considered. The lowest decrease in the tensile properties is observed for sample no. 6, i.e., 2% (warp) and 19% (weft). No major influence of freezing on the mechanical properties of the considered samples is observed.

Deformation and strain decrease with the increasing of the tensile strength of a material and/or decreasing of pressure acting on a material. The lowest deformation and strain values were achieved for material number 2 not subjected to the aging, and materials no. 2 and 6 subjected to the flexing damage. The opposite is noticed for fabric no. 1 subjected to UV degradation.

Safety factors of the considered materials not subjected to degradation range between 3.94 and 6.00. The safety factor of fabric no. 1 subjected to the UV degradation is equal to 1.33. Moreover, the obtained maximal strain is greater than the elongation at break of this material (8.5%). Although the implemented overload is very high and the maximal obtained values of strain rarely occur when distributions are considered, a paraglider covered with an UV degraded material no. 1 should not be allowed to be used.

The methodology described in this research can help to predict the paraglider covering materials’ behavior in flight; it assumes many cases, i.e., applying a new material or the material at any point of its life cycle. Based on it, an initial assessment of the safe usage of a considered material that was subjected to external factors can be performed. This method can be developed to become an optimized and easy tool. Thus, the practical implications of this model supported by numerical methods may result in saving time and cost in producing prototypes, as well as potentially assessing the safety of used wings.

Future research activity can introduce the application of different elastic–plastic damage models to determine the paraglider behavior during collapse. The problem was previously developed for rock material but is also applicable in composites [39]. The elastic–plastic damage model used to describe the degradation of the fiber-filling matrix is non-linear with progressive damage and inelastic strains. The other models, for example the hydraulic–mechanical–chemical damage coupling and modeling, can also be applied [40,41,42,43]. Some numerical codes allow to introduce the generalized description of thermal–hydraulic–mechanical–chemical coupling [44].

Another possible research activity can be the application of some unconventional methods to determine the optimal paraglider structure, cf. the lattice Boltzmann method [45], genetic algorithm [46] and neural network [47].

## Figures and Tables

**Table 1:** Basic characteristics of the analyzed materials.

Sample	Mass (g/m^2^)	Thickness (mm)	Max. Force during Elongation (daN)		Elongation at Break (%)	
			Warp	Weft	Warp	Weft
1	34	0.05	34	32	21	20
2	42	0.07	35	35	26	25
6	38	0.09	38	33	26	25

**Table 2:** Structural characteristics of the considered fabric samples.

Sample	Filament Diameter [µm]	Thin Yarn Diameter [µm]		Thick Yarn Diameter [µm]		Thin Yarns/ 1 Edge		Thick Yarns/ 1 Edge	
		Warp	Weft	Warp	Weft	Warp	Weft	Warp	Weft
1	15.15	128.8	152.3	187.9	225.9	22	18	2	2
2	24.77	160.2	198.8	202.4	255.8	20	15	2	2
6	20.23	159.4	224.9	220.0	322.4	20	15	2	2

**Table 3:** Air permeability values representing the considered fabrics subjected to aging.

Sample No.	Aging Method	200 Pa	1500 Pa	2000 Pa	2500 Pa
1	heating 50 °C 24 h	0.000	0.000	0.000	0.053
	heating 50 °C 48 h	0.000	0.000	0.000	0.057
	heating 50 °C 72 h	0.000	0.000	0.000	0.053
	heating 60 °C 24 h	0.000	0.000	0.000	0.060
	heating 60 °C 48 h	0.000	0.000	0.000	0.053
	heating 60 °C 72 h	0.000	0.000	0.000	0.060
	heating 70 °C 24 h	0.000	0.000	0.000	0.058
	heating 70 °C 48 h	0.000	0.000	0.000	0.059
	heating 70 °C 72 h	0.000	0.030	0.043	0.070
	Freezing	0.000	0.030	0.040	0.059
	UV	0.000	0.040	0.059	0.077
	flexing damage	0.050	0.685	0.865	1.125
2	heating 50 °C 24 h	0.000	0.000	0.000	0.125
	heating 50 °C 48 h	0.000	0.000	0.000	0.103
	heating 50 °C 72 h	0.000	0.000	0.000	0.107
	heating 60 °C 24 h	0.000	0.000	0.000	0.103
	heating 60 °C 48 h	0.000	0.000	0.000	0.107
	heating 60 °C 72 h	0.000	0.000	0.000	0.100
	heating 70 °C 24 h	0.000	0.000	0.000	0.118
	heating 70 °C 48 h	0.000	0.000	0.000	0.108
	heating 70 °C 72 h	0.000	0.050	0.090	0.270
	Freezing	0.000	0.055	0.080	0.117
	UV	0.000	0.083	0.310	0.430
	flexing damage	1.120	2.655	3.125	3.692
6	heating 50 °C 24 h	0.000	0.000	0.000	0.083
	heating 50 °C 48 h	0.000	0.000	0.000	0.075
	heating 50 °C 72 h	0.000	0.000	0.000	0.085
	heating 60 °C 24 h	0.000	0.000	0.000	0.105
	heating 60 °C 48 h	0.000	0.000	0.000	0.125
	heating 60 °C 72 h	0.000	0.000	0.000	0.105
	heating 70 °C 24 h	0.000	0.000	0.000	0.095
	heating 70 °C 48 h	0.000	0.000	0.000	0.093
	heating 70 °C 72 h	0.000	0.057	0.088	0.120
	Freezing	0.000	0.045	0.298	0.090
	UV	0.000	0.042	0.060	0.277
	flexing damage	2.050	7.200	9.500	11.600

**Table 4:** Tensile test results of the samples subjected to aging.

Sample No.	Direction	[-]		Heating 70 °C, 72 h		Freezing		UV		Flexing	
		F [N]	Ԑ [%]	F [N]	Ԑ [%]	F [N]	Ԑ [%]	F [N]	Ԑ [%]	F [N]	Ԑ [%]
1	Warp	340	21.2	318	23.2	400	30.6	121	10.3	339	21.0
	Weft	325	19.7	270	20.3	351	29.1	80	8.5	321	22.7
2	Warp	470	30.5	429	27.9	459	31.6	306	17.3	466	22.4
	Weft	465	29.9	425	27.4	451	30.4	224	16.3	402	22.4
6	Warp	269	20.6	258	19.3	251	18.6	263	21.1	260	20.1
	Weft	285	22.7	255	19.9	293	23.2	231	18.1	254	16.8

**Table 5:** The obtained Young’s modulus values.

Sample	E (MPa)	
	Warp	Weft
1	800	644
2	799	653
6	378	355
1 (heating)	748	625
2 (heating)	736	611
6 (heating)	370	342
1 (freezing)	805	800
2 (freezing)	757	541
6 (freezing)	333	370
1 (UV degradation)	355	309
2 (UV degradation)	642	357
6 (UV degradation)	422	333
1 (flexing)	798	652
2 (flexing)	690	623
6 (flexing)	372	417

**Table 6:** Pressure difference changes on the upper and lower surfaces of the considered materials in the symmetry plane (when maximal load factor is applied).

Samples: 1, 2, 6								
Distance from the Leading Edge	0.125 m	0.500 m	1.000 m	1.500 m	2.000 m	2.500 m	2.750 m	3.000 m
Upper surface	1047 Pa	991 Pa	879 Pa	772 Pa	672 Pa	560 Pa	476 Pa	392 Pa
Lower surface	420 Pa	560 Pa	532 Pa	504 Pa	487 Pa	465 Pa	420 Pa	392 Pa
**heating, freezing, UV; samples no. 1, 2, 6**								
Upper surface	1025 Pa	980 Pa	857 Pa	756 Pa	644 Pa	560 Pa	476 Pa	392 Pa
Lower surface	411 Pa	566 Pa	546 Pa	515 Pa	508 Pa	465 Pa	418 Pa	392 Pa
**Flexing damage, samples no. 1, 2**								
Upper surface	999 Pa	979 Pa	850 Pa	751 Pa	640 Pa	560 Pa	474 Pa	386 Pa
Lower surface	415 Pa	560 Pa	520 Pa	499 Pa	486 Pa	455 Pa	419 Pa	386 Pa
**Flexing damage, sample no. 6**								
Upper surface	700 Pa	677 Pa	581 Pa	438 Pa	287 Pa	157 Pa	72 Pa	28 Pa
Lower surface	336 Pa	464 Pa	433 Pa	350 Pa	260 Pa	156 Pa	71 Pa	28 Pa

**Table 7:** The minimum, average and maximum values of stress, strain and deformation of the considered cases.

Sample	Stress [Pa]			Strain [%]			Deformation [m]		
	min.	av.	max.	min.	av.	max.	min.	av.	max.
1	0.000	4.501 × 10^6^	2.934 × 10^7^	0.0	1.0	7.2	0.000	0.009	0.038
2	0.000	3.598 × 10^6^	2.220 × 10^7^	0.0	0.9	6.4	0.000	0.008	0.033
6	0.000	2.400 × 10^6^	1.555 × 10^7^	0.0	1.0	7.2	0.000	0.009	0.037
1 (heating)	0.000	4.425 × 10^6^	2.878 × 10^7^	0.0	1.0	7.2	0.000	0.009	0.038
2 (heating)	0.000	3.502 × 10^6^	2.185 × 10^7^	0.0	0.9	6.6	0.000	0.008	0.034
6 (heating)	0.000	2.388 × 10^6^	1.551 × 10^7^	0.0	1.1	7.3	0.000	0.009	0.038
1 (UV degradation)	0.000	3.578 × 10^6^	2.42 × 10^7^	0.0	1.5	9.1	0.000	0.012	0.048
2 (UV degradation)	0.000	3.311 × 10^6^	2.191 × 10^7^	0.0	1.0	7.5	0.000	0.009	0.040
6 (UV degradation)	0.000	2.464 × 10^6^	1.608 × 10^7^	0.0	1.0	7.2	0.000	0.009	0.038
1 (freezing)	0.000	4.555 × 10^6^	2.880 × 10^7^	0.0	1.0	6.8	0.000	0.009	0.035
2 (freezing)	0.000	3.521 × 10^6^	2.219 × 10^7^	0.0	0.9	6.7	0.000	0.008	0.035
6 (freezing)	0.000	2.464 × 10^6^	1.608 × 10^7^	0.0	1.0	7.2	0.000	0.009	0.039
1 (flexing)	0.000	4.452 × 10^6^	2.872 × 10^7^	0.0	1.0	7.0	0.000	0.009	0.037
2 (flexing)	0.000	3.428 × 10^6^	2.099 × 10^7^	0.0	0.9	6.5	0.000	0.008	0.033
6 (flexing)	0.000	1.928 × 10^6^	1.217 × 10^7^	0.0	0.9	5.6	0.000	0.008	0.033

## Data Availability

Data sharing not applicable.
